# American Dental Association

**Published:** 1892-11

**Authors:** 


### THE DENTAL REGISTER.

VOL. XLVI.] NOVEMBER, 1892. [No. 11.

### PROCEEDINGS.

### American Dental Association.

The thirty-second annual meeting of the American Dental Association was held at Niagara Falls, N. Y., Tuesday morning, August 2d, 1892.

The Association was called to order by the President, Dr. W. W. Walker, of New York, at 11:15 A. M.

After the usual routine business was disposed of the President read the annual address, of which the following is a synopsis:

After congratulating the profession on the advance made by it during the year past, the address drew attention to the coming meeting of the World's Columbian Dental Congress, and predicted a great advance to be made during the coming year in expectation of this event. The address urged that every member of the profession do all in his power to further the interests of this meeting in a professional as well as a scientific way, and as the estimated expense of the Congress would probably reach $30,000, he urged a generous co-operation in a financial way.

In regard to dental education and legislation, the address recommends the granting of diplomas from colleges only where the final examinations have been held by the regular State Examining Boards, and that then the diplomas of the various colleges could be recognized and accepted in the various States without question.

It was also desirable to adopt some plan for bringing the local and national societies into closer relationship, and the suggestion is made that a synopsis of all papers read at local societies should be referred to the proper section of the American Dental Association, which should be the custodian, and from these a report by the Secretary of each section could be made each year that would represent the progress of the profession. The suggestion is also made that the Association could prepare a list of subjects which could be sent out and be discussed in various societies, and these discussions, when collated and sifted would result in large additions to our knowledge.

The report suggests that only a business meeting be held next year, as the World's Columbian Dental Congress will probably absorb all the time of the members.

Before closing the address the President called attention to the death of Dr. John Allen, one of the oldest and widest known members of the profession.

The consideration of the address was referred to a committee consisting of Drs. E. C. Kirk, L. D. Shepard, and J. N. Crouse to report at a subsequent session.

Adjourned.

### TUESDAY EVENING SESSION.

### Report of Section VII.

ANATOMY, PATHOLOGY AND SURGERY, BY TRUMAN W. BROPHY, M. D., D. D. S.

This report consisted of an enumeration of a large number of contributions to medical and dental literature on the subjects of this section, which have appeared or been published during the past year.

The chairman also reported the following papers to be read at this meeting:

"Report of a Case in Practice of Pyorrhœa Alveolaris," by J. E. Cravens, D.D.S. "A New Operation for the Ex-Section of the Inferior Dental Nerve," by M. H. Cryer, M.D. "The Grinding Teeth of the Herbivorous Mammalia," by A. H. Thompson. "A Study of the Molar Teeth of the Proboscidians," by W. C. Barrett, M.D., D.D.S.

### PYORRHŒA ALVEOLARIS—A CASE IN PRACTICE.

Reported by J. E. Cravens, D.D.S., Indianapolis.

Patient, male, aged 50 years, apparently in good health, except catarrhal affection of nose and throat.

DIAGNOSIS—Gums red and angry-looking, bleeding easily and freely, considerable pus about incisors, and gum festoons swollen and of a violet color. Patient complained of heat and pricking pains in the teeth specified above; well defined pockets on the approximal surfaces, some of which were notably profound.

The treatment consisted in the thorough removal of the incrustations, and trimming the affected alveolar margins. The pockets were then thoroughly washed with hot water, and an application of a ten per cent. solution of commercial sulphuric acid made. The patient was directed to use powdered sulphur as a dentifrice morning and evening and avoid the use of soap and alkaline mouth-washes. This treatment was continued for three days, at which time there was no material change in the condition except a little less congestion.

The treatment was then changed. The pockets were washed clean with hot water as before and a ten per cent. solution of nitrate of silver was substituted for the sulphuric acid. After twenty-four hours there was a marked change for the better in the appearance of the gums and an appreciable diminution in the quantity of pus discharged.

This treatment was continued for four days and resulted in complete suppression of the discharge of pus, the congestion had all disappeared, and the pockets had evidently filled up by adhesion.

The essayist thinks the cure a good and permanent one. There was no painful or deleterious effect caused by the use of the nitrate of silver solution at any time. He attributes the cure to the use of the hot water and the astringent and antiseptic effect of the silver nitrate. The preliminary surgical treatment and also the detergent effect of the sulphuric acid were valuable factors in the rapid and satisfactory termination of the treatment.

DISCUSSION.

Dr. W. C. Barrett said there seemed to be no disease that came within the province of the dentist that calls for so much thought, study and careful investigation as pyorrhea alveolaris. He said that we have had but few cases of failure recorded in societies and elsewhere, yet our own cases were almost invariably failures. As to whether the cause of the disease was local or constitutional was not yet determined, but he had not been satisfied with any treatment he had yet tried, and he had tried about everything that had been recommended.

By local treatment he had been able to retard or arrest the progress of the disease, but had not been able to effect a radical cure. He could recall cases that had been under his care for years and years. Some of these cases would appear to be coming under control; the pockets would become almost filled with granulations and a cure seemed certain, when much to his dismay he would find the whole thing breaking down again and a speedy return of the disease.

The remedy Dr. Cravens had employed was a cauterant, and there was no therapeutic effect further than the cauterizing. From the case healing so rapidly it appeared to him that it was a case with local manifestations, and it was a question in his mind whether there would not be a return of the disease in three or six months. It will be interesting to watch the case and determine whether the result will be permanent or only temporary.

Dr. M. L. Rhein said it seemed to him that the case cited was not one of true pyorrhea alveolaris, but perhaps had its origin more from lack of due attention to the mouth. The essayist says that he thought the patient was in fairly good health. It is important to know the exact state of a patient's health, and the only way to determine this fully is by an examination both physical and chemical. The examination of the urine was important. In regard to the etiology of the disease he thinks that it is either a sequence of some constitutional disturbance or that disturbance is present at the time. If it be from disturbance of a disease that is hard to cure, as phthisis, etc.; the pyorrhea is hard, if not impossible, to cure; on the other hand, if it be from disturbance of a disease that can be eradicated, then a cure of the pyorrhea may be effected.

Many cases had passed through his hands that had not been cured, and from the condition of the system he knew that it was a hopeless task to undertake it. A thorough examination of the patient is therefore essential, and if disease be found it will assist us in our prognosis of the case and usually determine for us whether the case can be cured or not by the character of the disturbing disease whether it be one curable or incurable.

Dr. W. H. Morgan thought that the disease was largely inherited. He had had patients where the disease seemed to run through the entire family from the father and mother down to the children of ten and twelve years of age. He did not know that he had ever permanently cured a case, but he believed that the treatment should be mostly constitutional, with perhaps radical local treatment. He further stated that the gentlemen seemed to think that it was an incurable disease. He said it was an error, for every case could be cured and permanently too; extract the teeth and nature will do the rest. [Laughter].

A. W. Harlan said there is no objection to the use of sulphuric acid in the strength indicated by the essayist as a preliminary treatment. It is not strong enough to do harm. In the commercial acid there is 13 per cent. of sulphuric acid and 87 per cent. of water. To obtain the strength indicated this is still further reduced ten times, so that it is too weak to do any harm to the teeth or soft tissues. Diluted sulphuric acid acts much better than the aromatic sulphuric acid, for the aromatic sulphuric acid does not dissolve the calcarious or serumal deposits from the teeth, while the diluted sulphuric acid will. Dr. Harlan dilutes the acid with cinnamon water instead of water.

He said that nitrate of silver was used in various conditions, for antral disease, etc., but not in so strong a solution as 10 per cent., for it is a powerful astringent and stimulant.

Hot water is good as a therapeutic agent, and something the majority of dentists fail to utilize.

He said it seemed hardly possible that a cure of pyorrhea could be effected in ten days. If the pockets were half the length of the root, as indicated by the reader, you could hardly get a formation of new tissue in that time. In his opinion pyorrhea alveolaris is a purely local disease, with many constitutional manifestations which are concomitant with and not the cause of the disease.

Dr. M. H. Cryer, of Philadelphia, then read a paper entitled "A New Operation for the Ex-Section of the Inferior Dental Nerve." In the discussion of which Drs. Morrison, Fillebrown, Brophy and others said they preferred operating from the inside of the mouth.

The next paper read was entitled the "Grinding Teeth of the Herbivorous Mammalia," by A. H. Thompson, D.D.S.

The paper drew notice to the apparently promiscuous arrangement of enamel, dentine and cementum in the hoofed quadrupeds and vegetable feeders, and to the fact that the arrangement exhibits the highest degree of specialization found in dentition. This arrangement, seemingly without regularity, is uniform in species, but differs with families, hence the value of a knowledge of difference in forms for identification of species. The Ruminants furnish the best examples of this high degree of specialization. There are marked differences in the fissures and deep markings of the different genera of Ruminants, the goat, sheep, antelope, ox, deer. elk, giraffe and camel. The hog, hippopotamus, horse and rhinoceros also very markedly in size and form of the crowns. The proboscidians have a peculiar transverse marking, as the mastodon and elephant. The rodentia also exhibit a special transverse marking of alternate enamel and dentine. The molar teeth of the higher mammalia have by a process of evolution from simple types become organs of beauty and mechanical efficiency. The mechanical effects of use have been the principal factors in the development of tooth forms. The variation in food and consequent variation in movements of the jaw to produce effective mastication has brought that change in the form of the glenoid cavity and condyle of the jaw. The simple up and down motion of the jaw has been changed to the lateral and forward and back, and a combination of all as in man. This necessitates a special arrangement of dental tissues. The character of the food supply is the great influence which has produced this wonderful modification and specialization in the grinding or molar teeth.

This was followed by "A Brief Study of the Teeth of the Proboscidians," by W. C. Barrett, M.D., D.D.S.

The paper referred to the manifest entire loss of any preserved specimens of extinct species through the lack of chances of preservation by sudden upheavals or other incidents in the formation of the earth in the earlier periods which would encapsule and so preserve them for our edification. But in the pachyderms, as they now exist, or have existed in comparatively modern times, modifications can be traced which are a sure indication of the gradual development of certain species through the lapse of cycles of time. Families, whose dental development seems to be widely separated, on close inspection are found to be close congeners. For instance, there is a distinct relation between the dentition of the tapir, with its forty-two teeth, and the elephant, with only six. The paper described the manner of the formation of the molar teeth of the elephant, the deposition of the various tissues, and models were shown to illustrate this development, the distribution of tissues being such as to, by wear, prepare the best possible masticating surface. The manner in which these teeth are worn out, cast off, and replaced by their successors throughout the entire life of the animal, was described and illustrated. These teeth are erupted in such a way as to best economize the tooth material and furnish the animal with a satisfactory dental organ for a long period. The time of eruption, the number of plates forming the crown, and the duration of each successive tooth, was shown to be regular and systematic, the first molar having a fewer number of plates and the least number of years, there being a gradual increase in each succeeding tooth of both plates and years of life. There are abnormalities in the development of the incisors or tusks, due, probably, to accident to the alveolar process. The celebrated elephant "Jumbo" had two molars in each jaw fully erupted, and none of the visible teeth occupied their normal positions. The probable reason why the two molars were present at the same time was due to the fact that the animal had always been in captivity and been fed on food free from gritty or wearing material, and the first tooth was not used up in time to be cast off before the full time for the emergency of its successor. This is the opinion of Prof. Flower of the British Museum. But Dr. Barrett thinks it due to some accident to the formative germs of the teeth.

Adjourned.

### WEDNESDAY MORNING SESSION.

Dr. Barrett, of Buffalo, N. Y. read a paper on "Comparative Dental Anatomy:" and Dr. W. B. Ames, of Chicago, read the report of Section 1. on Prosthetic Dentistry, Chemistry and Metallurgy, both of which were fully discussed.

Dr. George Evans, of New York, then presented a new method of making Crowns, the discussion of which was as follows:

Dr. J. D. Patterson asked how the doctor controlled the case so as not to melt the crown and yet get the proper amount of heat to fuse the enamel.

Dr. Evans said the gold was alloyed with platinum. While baking, the open end of the muffle is turned toward the operator so that he can watch the case as closely as necessary. He advised those first attempting the work to experiment to determine how high to carry the heat without injuring the case.

The enamel could be fused by a blow-pipe flame, but it is better to use the muffle to protect the case from carbon.

Question—What proportion of platina do you use?

Answer—From 3 per cent. to 4 per cent. for crowns.

Dr. McKellops said he saw the same operation some years ago at Berlin, Germany. It was brought out by Dr. Cunningham. He presented specimens of various colors, such as gum color, etc., but said the formula for the enamel was secret and he would not disclose it.

Dr. Evans said that he used enamel made by Timme & Co., of New York. He further stated that he claimed no originality for this work; that he obtained his ideas of a muffle and of glass enamels from Dr. Herbst.

Dr. Guilford said that when a brother of Dr. Herbst was in America, attending lectures at a Philadelphia college, he demonstrated the using of pulverized glass on the teeth to imitate the gum in color, etc. To make a muffle that has been very satisfactory in his hands Dr. Guilford takes a suitable piece of platinum of 32 to 34 gauge, bends to shape over the fingers, and flows pure gold over the joints to unite the parts. He further stated that if you have a plate tooth and want to make the pins headed for rubber or other use, file off the pins, place a particle of borax on the end of each pin, lay across both pins a piece of gold plate, lay the tooth on asbestos paper, place in the crucible, apply heat until the pieces unite. There is no danger of checking the tooth as the air in the muffle heats quicker than the tooth.

In regard to glass fillings, he said that we get on very dangerous ground when we attempt them, for the glass lacks that quality of strength necessary for the restoration of tooth-corners and in other locations where there is leverage on the filling. Besides such fillings bulge, and it is almost impossible to secure the proper shades.

Dr. McKellops: The originality of enameling belongs to the watch-makers.

### Report of Section II.

### DENTAL EDUCATION, LITERATURE AND NOMENCLATURE, BY LOUIS OTTOFY, D.D.S.

*Education.*—There are 38 dental colleges now in existence, 5 of which were organized during the past year. 1483 students were graduated in 1892, the Philadelphia School leading with 148, the Meharry and German American having one each; 851 were graduated from 10 colleges. The lengthening of the college course does not seem to have materially decreased the number of students entering dental colleges.

There are 130 local societies now in the United States, with a membership of 5,000; 24 of these societies were represented by delegates in this association last year.

The reading scheme in the post graduate course is now under way and much interest seems to be awakened in a two year's reading course in dental anatomy and crown and bridge-work. Other courses on dental science and art are to be provided in the near future.

*Literature.*—The dental periodicals show a marked improvement in the quality of the matter they contain. The Illinois Dental Society advises the establishment of a weekly dental journal, as the monthlies are too slow in getting the *news* to their readers; it was also here advocated that a quarterly journal, publishing only original matter for which $5.00 per page had been paid, would meet with financial and professional support.

Permanent dental literature has been enriched by a revised edition of Harris' Dental Dictionary; a new edition of Sewill's Dental Surgery; the fourth edition of Gorgas' Dental Medicine; Charts of Typical Forms of Irregularities, by Eugene S. Talbot; a second edition of Black's Dental Anatomy; Catching's Compend for 1891, and last, a new work on Dental Jurisprudence, by Wm. F. Rehfuss, D.D.S., of Philadelphia.

In dental nomenclature there is no report to make.

---

### WEDNESDAY EVENING SESSION.

The report of the Committee on the President's Address was read by Dr. L. D. Shepard, of Boston, and was as follows:

Recognizing the importance of the World's Columbian Congress at Chicago in 1893, we recommend that an appropriation of $500 be made from the funds of this Association to the treasury of the World's Dental Congress. The Southern Dental Association has already made a similar appropriation.

In regard to dental education and legislation your committee respectfully report that they are of the opinion that the State Examining Boards should be appointed by the Governor of each State upon the recommendation of the members of the various State and local societies; and we believe that all such matters should be managed in each State by the State Dental Society, which should practically embrace the whole reputable profession of the State. This we also regard as the principal factor in securing the best laws and their proper enforcement, the most competent Board, the much desired inter-State, county and general recognition of State licenses.

In regard to the formation of a committee on State and local organizations, we would offer the following resolutions:

That a Standing Committee, composed of three members, shall be appointed originally for the terms of one, two and three years respectively, and that all vacancies due to expiration of the term thereafter shall be filled for the term of three years. All vacancies occurring from any other cause shall be filled for the unexpired term.

The basis of the plan and scope of work of said committee, and its duties in relation thereto, shall be suggestions as set forth in the address of the President; also the articles from which he quotes and in harmony with the circular letters issued by the Chairman of the Executive Committee.

In regard to the meeting of the Association for next year, we offer the following resolution:

*Resolved*, That the annual meeting of this Association for 1893 be convened at the time and for the purpose suggested in the President's Address, and that the consideration of the report of the Special Committee embodying certain proposed amendments to the Constitution and By-Laws, be laid over for consideration at that meeting.

We also recommend that the matter of the death of Dr. John Allen be referred to the Committee on Necrology for appropriate action.

Respectfully submitted,

Edward C. Kirk,
L. D. Shepard,
J. N. Crouse.

### Report of Section III.

### OPERATIVE DENTISTRY, BY A. W. M'CANDLESS.

During the year there have been published 60 articles, in different dental journals, on operative dentistry, mostly on filling materials and treatment of pulpless teeth. Only one article on conservative treatment of the pulp, indicating that this subject is not a popular one, or that the practice is not sufficiently successful to inspire any one to write about it. This is at variance with the report from this section last year. The effort to get reports of local society transactions for this meeting failed to bring satisfactory responses, but it is hoped by more persistent pushing of this matter next year will meet with more definite and favorable replies.

Some progress seems to have been made in the application of agents for painless operations on the pulp.

The report mentions as new appliances and helps in operative work Dr. W. B. Ames new oxyphosphate of copper, crystal mat gold, Dr. Keefe's improved rubber dam clamp, improved flexo files, apparatus for softening gutta-percha, dento electric cautery, improved root dryer, cotton pellet roller, root trimmers, Bosworth mallet, silver and platinum alloy, screw posts and hand matrix for manipulating plastic fillings.

### REPLANTING AND TRANSPLANTING TEETH, BY W. N. MORRISON, D.D.S.

The essayist stated that of the cases reported to this association in 1875-6 two of the replanted ones are still in the mouth, in good condition; one of them being in the mouth of a person with most unfavorable constitution.

The essayist complained of the unfavorable comment of the profession in regard to this operation as calculated to hinder progress and to throw difficulties in the way of those who were practicing this operation with sufficient success to justify it. The essayist enumerated a number of successful operations, and although he experiences failures, he believes the operation of sufficient durability to warrant its use in many instances where extraction and artificial substitution would be unsightly or undesirable.

One of the important elements of success in this operation is the attainment of absolute rest, or freedom from motion of the implanted tooth. This is accomplished best by a figure of eight suspensory copper wire ligature from the crowns of adjoining teeth over or under, (as the case may be), the cutting edge of the implanted tooth, also securing the ligature in position, and the interdental spaces with phosphate cement, and this is worn for six or eight weeks.

DISCUSSION.

Dr. Head asked how many replanted teeth lasted five years?

Dr. Morrison: Not to exceed twenty per cent.

Dr. Darby: An inquiry of this kind may be misleading to the younger members of our profession by leading them to think that implanted teeth will last but a short time. I have seen many of these teeth that have done good service for six or seven years and are still in good condition. When Dr. Younger was east, some years ago, he implanted in one case four bicuspids and two central incisors. For about six years they did good service but finally one of the centrals was lost. Having the patient in charge I inserted a temporary plate, which was worn a few months, then I drilled a new socket and implanted another tooth. The tooth became firm after a few months time and is firm to-day.

Altogether I have myself implanted thirty-six teeth out of which I have had but five failures. Some of the teeth have now been in position four or five years.

There are places where this operation is a valuable one; for instance, to supply the space of a lost lateral or central incisor. If the tooth implanted lasts only four or five years it is worth the operation and another can be implanted. I have engrafted artificial crowns on roots of teeth and implanted when I could not find a natural tooth of the right shade. I hope the profession will not give up this valuable operation, for there is certainly merit in it and I, for one, should dislike to see it get a "black eye."

Dr. W. H. Morgan: Bony ankylosis was spoken of as one of the forms of attachment of implanted teeth; there is no such thing as a bony ankylosis; it is the uniting of cartilage and I think the term is improperly applied. The tooth is held by encystment, cartilagenous or otherwise, but never by a bony union; the dead and living will not unite.

Dr. L. Ottofy said it was unfortunate that Dr. Younger, when east, had to implant teeth in all sorts of mouths, favorable and unfavorable alike. One great objection to the implantation of teeth is the scarcity of perfect teeth. I have lost several cases on account of checks, etc., in the implanted tooth. I almost invariably cut off the crown of the tooth, adjust a Logan crown on the root using a layer of gold between root and crown for protection.

Some of the teeth I have implanted have been in the mouth for five years. Most of the first cases I tried were failures on account of not taking proper care in the selection of patients. On the other hand there is hardly a case where the patient was selected but is in good condition to-day. How long implanted teeth will last we cannot say further than judging from the past. Some of those implanted by Dr. Younger several years ago are still firm. From what we know now I would say they would last from four to seven years.

MATRICES, BY J. E. CRAVENS, D.D.S., INDIANAPOLIS, IND.

Dr. Cravens said: It is a test of an operator's judgment sometimes to determine whether he shall use a matrix or merely a support for his filling material. There is a wide difference. * * * A matrix for filling should possess certain advantages, such as thinness for economy of space, close conformity to cavity margins, however irregular; sufficient malleability to enable the operator to bend and dilate at any part and to any extent desirable; it should be cheap enough to be thrown away after once used. * * * A writer in a dental journal declares that perfect gold filling cannot be done in matrices. I think him mistaken, for every cavity is a matrix so far as it effects the form of its contents, but from cases observed I am satisfied that failures may easily be made and that an operator's confidence in matrices sometimes is abused, because failures at obscure margins may not be discoverable until after the completion of the work. The usual fault with matrix filling consists in imperfect adaptation of the filling material at cervical and lateral margins. Gold cannot always be depended upon for perfect adaptation at these points unless in excess, and close matrices do not admit of excess.

Dr. Cravens thinks a support, a piece of thin metal of a springy, non-malleable quality placed between the teeth, with no attempt at conformity to cavity margins, is preferable. The margins should stand free, particularly in filling with gold, so that an excess may occur and better adaptation be derived. He recommended the construction of matrices of German silver, cut and fitted to the curves of the gum in the same manner as fitting bands for gold crowns. A band closed with a lap joint next to the cheek and fastened with soft solder may be dilated or bulged at any part, the metal yielding readily to a moderately firm pressure, so as to conform to an approximating tooth or give fullness at any point. Amalgam thus supported may be left several hours to harden and often accommodate the dentist's demands. For gold plain shields will hold all the space required, and if a little more space should be required for finishing purposes it may be readily gained during the operation by driving the gold against the shield, thus actually wedging the teeth slightly apart.

DISCUSSION.

Dr. J. A. Swasey prefers a matrix made of brass. He does not want any obscure walls, but wants to see every portion of the cavity. He said a man who does not wedge a tooth and fit a matrix so that he can see all portions of the cavity is not an operator. Making a cavity a simple one is the object of a matrix.

Dr. McKellops said the teeth must be separated so that the operator can see every portion of the cavity. He does not think a good filling can be made if any of the walls are obscure. If a tooth is tender or sore from wedging he uses separators to hold it firm so that there is little or no discomfort from malleting. He thinks the object of every operator should be to relieve the patient of all pain possible.

Dr. J. Taft said there was a great difference in opinion regarding the use of the matrix. It was a useful article if properly used, but much faulty work was done with matrices. He uses the partial matrix, covering one-fourth, one third or one-half of the cavity, where the cavity is such that he can see into it better by so doing. When he has filled nearly to the top of the matrix it is removed and the filling completed without one.

Dr. W. H. Morgan wished to challenge the idea that cavities could not be successfully filled unless the whole cavity could be seen by the operator, for he had himself made successful fillings where only a portion of the cavity could be seen. He uses non-cohesive gold, testing its solidity by the touch.

Dr. McKellops said that pure gold was always cohesive; there was really no pure non-cohesive gold. He said, of course a cavity could be prepared with the aid of the mouth-mirror, but you cannot make the operation perfect unless you can see every portion of the cavity. At least it takes a good man to do it.

Dr. Morgan: That is why I can do it. I can prove my assertions with any amount of specimens.

Dr. Johnson asked Dr. Allport to speak to the society about non-cohesive gold.

Dr. Allport said he supposed the gentleman meant the relative merits of the two. Each has its place, and neither can take the place of the other. We need both. He said to go back forty-seven years they had but one kind of gold—the non-cohesive. After using this it seldom happened that there was discoloration around the filling when made by a good operator, but the greatest objection was that the fillings scaled and the portions were thus lost. Next came the sponge gold which was all cohesive. This was just what was needed to use over the non-cohesive gold to protect the surface of the filling. Everybody lauded it and used the new gold because it would stick together. But there was soon a revelation; the teeth turned blue under these fillings. At the time it was thought it was due to impurities, but it was not so, but because the plug did not fit the cavity. It is the same to-day with cohesive gold and the majority of operations are not as permanent as when non-cohesive gold was used. The trouble is that the profession is out of the old way of operating. You cannot do with the mallet what can be done with hand pressure. By the use of the mallet many teeth are unnecessarily destroyed. There is a tendency of the gold to draw away from the cavity walls when condensed in the centre of the filling. He said he had filled hundreds of teeth without seeing into the cavity with even a mirror except when it was excavated.

Dr. Brophy said the idea of the matrix originated with Dr. Dwinelle some thirty-five years ago. When thoroughly understood the matrix is a good thing, but when it is not it is worse than none.

### THURSDAY MORNING SESSION.

Section IV, on Histology and Microscopy, made its annual report through its Chairman, Dr. Frank Abbott, of New York.

Dr. C. W. Stainton, of Buffalo, N. Y., read a paper entitled "Crownless Teeth."

The case presented was of three members of a family of six, showing an unusual hereditary occurrence of the absence of the crowns of their teeth.

The history is that the grandmother had such teeth and it was inherited. No history back of this could be traced. The father had poor teeth, inherited from his mother, the teeth being the poorest of the family of eight and entirely crownless. The models presented an appearance of the teeth having been cut off almost to the gum line. The peculiar formation cannot be accurately described without showing the models which will be fully illustrated in the transactions of the society.

### Report of Section V.

Section V, on Materia Medica and Therapeutics, made its annual report through the Secretary of the Section, Dr. Geo. E. Hunt, of Indianapolis. Among the remedies described were Pental, Aristol, and Bichloride of Mercury.

A. W. Harlan read a paper on "Europhen, and Trichlor Acetic Acid."

Europhen is a yellow amorphous powder made by the action of iodine, obtained from iodine of potassium, upon isobutyloresol.

The average per cent. of iodine in the compound is 27.6. The powder is resinous to the touch and adheres tenaciously to wounds and the mucous membrane. It has a specific aromatic odor like saffron, which is not perceptible in mixtures or solutions. It is insoluble in water or glycerine, but readily soluble in alcohol, ether and chloroform, and solution made by the agents, it is also readily soluble in the fatty oils. Europhen in contact with water, such as the secretions of wounds, etc., gives off slowly small quantities of iodine, which immediately re-combine to form a soluble iodine compound. While alcoholic solutions give up iodine readily, the ether solutions give it off more freely. Mixtures with zinc oxid and mercuric oxid should be avoided. Lanolin mixtures are very desirable, as the lanolin takes up a great deal of water, and thus favors the formation of the soluble iodine compounds. All solutions should be made and kept in low temperature, free from light and moisture. Europhen when applied to wounds does not cake as does iodoform, neither does it produce toxic effects; it is not poisonous. Fifteen grains have been administered with no bad effects. When applied to inflamed gingival conditions, caused by the insertion of crowns or bridges, the soreness and irritation promptly subsides. It is also beneficial as an application to syphilitic ulcerations and irritations about the mouth; promptly allays the pain of an inflamed or suppurating pulp, the lanolin preparations being used in these cases. Take of lanolin 75 parts, of europhen 25 parts. This also makes a useful preparation to apply to an inflamed sore mouth caused by wearing artificial dentures. It will doubtless prove a useful dressing in the powder form for empyemia of the antrum. On account of the lightness of the powder it is peculiarly adapted to insufflation.

Acidum Chloraceticum—Trichlor Acetic Acid (C. 2; H. Cl. 3; O. 2). This is made by treating chloral hydrate with three times its volume of nitric acid and subjecting the mixture to the rays of sunlight until the red fumes disappear; this is then distilled and the portion coming over at 195° C. is pure Trichlor Acetic Acid. It is a colorless crystalline substance, and readily soluble in water or alcohol. It is a powerful caustic, readily destroying the mucous membrane or epidermis. A three per cent. solution is a local stimulant—an astringent; a ten per cent. solution should be used to decalcify or remove the serumal deposits from the roots of teeth in the treatment of pyorrhœa alveolaris and kindred diseases. As a cauterant agent, it is useful in destroying the pyogenic membrane in pus cavities; also for the destruction of morbid growths, hypertrophied or excessive tissue. A $\frac{1}{2}$ to 1 per cent. solution makes an agreeable refrigerant mouth-wash.

### SYRUP OF IRON CHLORIDE.

In all cases where an efficient preparation of iron is to be used as a tonic, the formula of Dr. Neld should be preferred. It is a non-alcoholic preparation, and as prepared by Parke, Davis & Co., it is not only an elegant but very useful preparation; no corrosive action of the mucous membrane or teeth is produced by it.

### DISCUSSION.

Dr. F. Abbott said he was astonished that Dr. Koch had made such a mistake and that so many others had committed the same mistake by the continued use of bichloride of mercury.

Bichloride of mercury will destroy human life and it seems that it must kill micro-organisms. He had seen what seemed wonderful results from the use of bichloride of mercury in the mouth and relied upon it to a great extent.

Eminent surgeons will tell you that bichloride of mercury can be relied upon all the time. If they get good results from its use it seems to me that there must be some mistake in the experiments of this man Mc Clintock.

Chloride of zinc he thinks one of the very best remedies for pyorrhœa alveolaris. It does just as well as sulphuric acid and acts more kindly.

Europhen.—If this remedy will relieve excoriations under plates, relieve the pain around roots of teeth, etc., it is certainly a remedy worthy of our consideration. Weld's Syrup if left to stand in an office for ten days or so will precipitate. Tincture of chloride of iron has done inestimable injury to the teeth, and if this remedy contains the original properties of the tincture, the precipitation overcome, and will not injure the teeth we have got a wonderful thing.

Dr. J. S. Marshall: It is a mistake to think that every eminent surgeon uses bichloride of mercury, for many use sterilized water instead for disinfecting instruments, hands, etc., and washing the parts before operating. With this they have had better results than with bichloride of mercury.

PENTAL.—With regard to this and other anæsthetics Prof. Turet, Germany, reports 109,230 administrations with 39 fatalities; chloroform was administered 94,123 times with 35 deaths; ether 8,432 times with 1 death; ether and chloroform combined 2,891 times with 1 death; ether and alcohol 1,380 times, 1 death; bromo ethel 2,179 times, no death; pental 219 times, 1 death, which will show the average of deaths greater from use of pental than any other anæsthetic.

Dr. Peabody described his method of vaporizing iodoform which was substantially the same as given in our report of the Mississippi State meeting.

Dr. G. E. Hunt: As Dr. Abbott has said, bichloride of mercury is sure death to animal life. It should be remembered, however, that the higher and more complex the organized being the greater will be the influence of deleterious agents, and as we go down the scale to the simpler forms of life we find more resistance to the effects of these agents. While bichloride of mercury is poisonous to animal life it has not been proven that it is death to vegetable. The ideas advanced by Prof. McClintock are new to me, but the experiments have been carefully made and there may be a great deal of truth in them. Iodoform vapor is nothing new; it was brought out by Dr. W. H. Whitslar five years ago. It is my opinion that the iodine is evaporated and what remains is simply a carbonaceous residue which is aseptic but not antiseptic.

Dr. F. Abbott: Years ago it was thought that carbolic acid was the best antiseptic ever known. One says it is the best, while another sees germs working their way around in it, etc. It was afterward shown that when properly diluted carbolic acid would destroy germ life. Why? Because germs live upon liquids, and if taken into the body it destroys. Now, it may be the same with bichloride of mercury, that in too strong solutions the micro-organisms refuse to take it, while in weak solutions, as $\frac{1}{20000}$ or $\frac{1}{10000}$ they take it and it kills. I use it in root canals in the strength of $\frac{1}{10000}$, but never stronger.

Dr. J. E. Low thinks iodoform is too disagreeable to have about the office, and he has had patients complain of tasting it for some months after its use. He does not think that a root can be perfectly disinfected by iodoform vapor; believes that phagocytes destroy the remainder of the germs.

Dr. H. H. Fuller said it was not the bacteria themselves that make the trouble but the dead tissue that forms food for the bacteria. In our operations if we get rid of obnoxious matter we get a cure. He thinks bacteriology has been carried too far, and that cleanliness is of more importance than the use of antiseptics.

Dr. C. N. Peirce uses Trichlor Acetic Acid on epulis tumors, spongy gums, growths over third molars, etc., applied by means of wedge-wood stick. For calcic pericementitis use on a spatula for cleansing roots, can use full strength if necessary. It has happy results on the tissues, being escharotic and astringent. It arrests pus accumulation in pyorrhœa alveolaris. For putrescent pulps force it into the root canals. It destroys the tissue and purifies in a few moments' time more perfectly than carbolic acid.

Dr. J. D. Patterson said he had been, for a few months, experimenting with the Stebbins' method of applying silver nitrate to cavities in deciduous teeth to check decay. He said if results were satisfactory it was certainly a good thing, and that the discoloration it caused was no objection if it only preserved the teeth.

Dr. J. Taft said that Dr. J. Taylor forty years ago recommended nitrate of silver for decay. He based his theory on the color of decay; that the black variety progressed more slowly than others, and as nitrate of silver turned tooth substance black it would probably retard the progress of caries. Since that time Dr. Taft has used it many times and knows that under favorable circumstances decay will be arrested. After its use if the mouth and teeth are not kept properly cleaned the effects will not be so lasting.

Dr. McManus said his preceptor used nitrate of silver at the necks of the teeth as treatment for sensitiveness, and that it was successful all but the black line. He knows that Dr. Stebbins' cases are successful.

Dr. Peirce said nitrate of silver had long been used as an obtundent, but for the arrest of decay he thought Dr. Stebbins was the first to introduce it and should have that credit.

Dr. A. W. Harlan said that the theory advanced regarding the action of bi-chloride of mercury was old. La Place was the first to point it out. Five parts of tartaric acid makes bichloride of mercury absolutely reliable as a destroyer of micro-organisms. Equal parts of peroxide of hydrogen and $\frac{1}{1000}$ sol. bichloride of mercury is effective. Europhen is an agent to take the place of iodoform ; it answers all the purposes of iodoform and is lighter and much more agreeable. Trichlor Acetic Acid is useful for serumal deposits, a ten per cent. solution in water, and as an astringent for the tissues. It makes a good mouth-wash in $\frac{1}{2}$ to 1 per cent. solution. The antidotes are bi-carbonate of soda or other alkaline salts.

### THURSDAY EVENING SESION.

### Report of Section VI.

The Report of Section VI, on Physiology and Etiology was made by the Chairman, Dr. H. A. Smith.

Dr. J. D. Patterson read a paper on " the Effect of the internal Administration of Certain Drugs upon the Tissues of the Oral Cavity," in which the essayist referred to the prevalent idea that the internal administration of mercurial and iron preparations were markedly harmful to dental structures, and suggested that these views, while having much of truth, were also elements of error in regard to their administration and the effects produced. The essayist desired to call attention particularly to the manifestations of mercury in the mouth when given internally, and cited the description of the physiological effects as seen in the mouth as described in Potter's Materia Medica, as an accurate statement of these manifestations.

The essayist then gave the result of some personal observations to prove that these manifestations should not always be taken as an indication of mercurial poisoning.

The characteristic "blue line" which every text book describes as present in mercurial stomatitis, and is given as one of the ever present symptoms of ptyalism, is in fact, due to the local accumulation of salivary calculi, and in every case can be obliterated by one operation, and in no case that has ever been brought to his attention has he failed to verify this statement.

As to the "inflamed and spongy gums" the author also declares that this condition is susceptible of control and cure by means of hygienic agencies, cleanliness and antiseptics, and this, too, without discontinuing the administration of the mercury to the patient except in cases where the condition is the result of syphilitic taint.

These statements he makes on the basis of a series of experiments extending over two years, under favorable circumstances in regard to abundance in the supply of cases.

The author concludes from this that these conditions are not essentially a result of the exhibition of mercury, but are caused by a lack of hygienic attention to the mouth when this drug is being administered. It does cause congestion of the gum tissue, also an increase of the salivary activities, and there is generally less care taken of the mouth and teeth at such times and consequently greater deposits may take place. This is also parallel to the conditions existing during pregnancy. It is lack of care and cleanliness that causes the destruction of the teeth of women during confinement and not, as is generally supposed, a withdrawal of lime salts from the teeth.

The essayist closes with the statement "that he believes that much of the supposed ravages of mercury upon the tissues of the mouth may be safely ascribed to pure carelessness, ignorance and neglect."

This paper was fully discussed by Drs. Fillebrown, Morgan, Hunt, Smith, Barrett and others.

The annual report on the "Condition of Prehistoric Crania" was read by Dr. John J. R. Patrick, of Belleville, Ill., which was discussed by Drs. C. N. Peirce, H. A. Smith, and Louis Ottofy.

The Committee on State and Local Organizations appointed by the president, consisting of the following gentlemen, viz.: E. C. Kirk, Philadelphia, chairman; J. N. Crouse, Chicago, and Louis Jack, Philadelphia, presented the following set of questions, which they recommended for discussion among the State and Local societies during the coming year:

No. 1. Should examining boards have power to grant certificates of qualification to undergraduates?

No. 2. Should immediate root-filling be practiced while purulent conditions exist at the apex?

No. 3. What are the best materials to enter into the composition of temporary fillings, to be retained for a minimum of three years?

No. 4. What are the best methods for obtunding sensibility of the dentine by either local or general means? Should arsenic ever be used?

No. 5. What are the best forms of partial lower dentures, and the methods for constructing the same?

No. 6. Corrective dentistry; its present status. What are the simplest and most universally applicable forms of apparatus and most efficient retaining fixtures?

No. 7. To what extent, and under what conditions is the collar crown a cause of pericemental inflammation?

No. 8. In cases of congested pulp, should the arsenical application be made without preliminary treatment?

No. 9. What are the advantages and disadvantages of the use of the matrix—1st, with gold; 2d, with plastics?

No. 10. The etiology of pus-formation?

### FRIDAY MORNING SESSION.

The election of officers was held during this session, which resulted as follows: President, J. D. Patterson, Kansas City,Mo.; First Vice-President, J. Y. Crawford, Nashville, Tenn.; Second Vice-President, S. C. G. Watkins, Mont Clair, N. J.; Corresponding Secretary, F. A. Levy, Orange, N. J.; Recording Secretary, Geo. H. Cushing, Chicago, Ill.; Treasurer, A. H. Fuller, St. Louis, Mo.

Executive Committee for three years, Drs. W. W. Walker, and S. G. Perry, of New York, and D. N. McQuillen, of Philadelphia.

After the installation of the newly-elected officers the Association adjourned to meet in Chicago, in August, 1893.

